# Professional Caregiver's View on Mental Health in Parents of Children with Developmental Disabilities: A Nationwide Study of Institutions and Consultation Centers in Japan

**DOI:** 10.5402/2012/121898

**Published:** 2012-01-11

**Authors:** Tomoka Kobayashi, Masumi Inagaki, Makiko Kaga

**Affiliations:** ^1^Department of Developmental Disorders, National Institute of Mental Health, National Center of Neurology and Psychiatry (NCNP), 4-1-1 Ogawa-Higashi, Kodaira, Tokyo 187-8553, Japan; ^2^Department of Pediatrics, Social Health Insurance Central General Hospital, Shinjuku, Tokyo, Japan

## Abstract

Parents of children with intellectual disabilities and/or physical disabilities are supposed to have an increased risk for parenting stress and psychological distress. We as professional caregivers sometimes experience difficulties in keeping good relations or communicating with the parents. Professional workers in 460 institutions and consultation centers throughout Japan answered a questionnaire on their clinical experiences. About 90% of the facilities experienced “distressed parents,” and the parents' condition such as mental health seemed to influence this. Signs of a depressive state were the most common psychiatric disturbances detected, and it was notable in the pervasive developmental disorder group. More welfare support, presence of support groups, support from other family members, and medical treatment of the parents' problems were considered to be helpful and thus requested to be improved. Training more professionals who can properly deal with the parents' mental health needs is an urgent matter that must be tackled.

## 1. Introduction

Many previous studies have revealed that parents, especially mothers, of children with developmental disabilities such as intellectual disabilities (IDs), developmental delay, and physical and sensory handicaps are more likely to show signs of psychological distress or depressive symptoms and to exhibit lower well-being than parents of typically developing children [1–5]. It is generally accepted that caring for a child who has a developmental disability may involve significant and prolonged periods of time and energy [6]. Since the majority of this increased daily care-giving burden for these children is carried by their parents, they are supposed to have an increased risk for high levels of stress, and thus some cases may be linked to depression [7, 8].

Recently, the mental health needs of adults with ID have also been taken up for discussion. Studies have revealed that psychiatric disorders are more prevalent in people with ID compared within the general population [9]. In fact, the burden of mental illness on health and productivity is tremendous [10]. Specialist psychiatric services for people with ID are available in some countries such as the UK and the USA; however, the provision of high-quality psychiatric services remains a major concern in many countries [11]. There have been few reports on mental health care from Asian countries including Japan [12]. Evidently, the current psychiatric services in Japan are not adequate to meet the complex mental health needs of people with ID appropriately, sensitively, and effectively. Failure to provide services that meet the needs of these individuals may lead to problems such as high-level caregiver stress and inappropriate therapy [13]. The paucity of trained psychiatrists and other allied professionals is a major barrier to the development of this subspecialty in many countries [14].

In Japan, high-quality psychiatric services for the parents of children with ID are assumed to be not well established. So far, the major portion of resources has been directed to the treatment of and services connected with the children, and not enough attention has been paid to the actual mental condition and needs of the parents. To our knowledge, there have been no reports on a nationwide scale on the mental health and distress of parents having children with developmental disabilities in Japan. Since the understanding of the parents' mental health may be essential for providing more appropriate support, we aimed to obtain a clear perspective on the situation facing the parents through this facility-based nationwide study.

## 2. Methods

The current survey included 1,102 institutions and consultation centers for children with developmental disabilities distributing in all prefectures in Japan, which were listed in the national register of agencies, organizations, and institutions related to intellectual disabilities [15] and the directory of support centers for individuals with developmental disabilities [16]. In Japan, these institutions and centers play a key role in social support networks. Professional workers in these facilities come into contact with children as their clients, and also with the children's guardians, mainly with the mothers, on a daily basis. A structured questionnaire was sent to the institutions and consultation centers. Professional workers working in these centers replied on their clinical experiences.

The questionnaire was written in Japanese and was divided into two sections with a total of 47 multiple choice questions. There was space to fill out for 5 of the questions. The first section sought information about the characteristics of the facility, such as staff adequacy, the scale of the facility, the health promotion services provided, the ages of their clients, and the clients' main disabilities, namely, ID, pervasive developmental disorder (PDD), profound intellectual and multiple disabilities (PIMD), physical disabilities, and others. The second section sought information about the clients' parents, such as signs of mental health distress and their needs, from the professional caregivers' point of view. Also measures taken by the professional caregivers when encountering distressed parents were asked in detail.

Questionnaires were mailed in October 2008, with an explanatory letter to the person in charge of each institution or consultation center. When agreement was met to participate in this study, one or more service providers in each facility completed the questionnaire based on all sources of information available. The questionnaire was to be returned unsigned, using the stamped, self-addressed envelope enclosed. For ethical considerations, the information disclosed in the questionnaire was in a form which made it impossible to identify the facility and clients. This study was approved and carried out under the supervision of the Japan League on Developmental Disabilities (JIDD).

The statistical analysis including the chi-squared test was performed with the aid of the Statistical Package for the Social Sciences 15.0 (SPSS Japan, Inc.). A two-sided *P* value of 0.05 or less was considered to be statistically significant. Answers to open-ended questions were analyzed thematically by coding them into separate categories.

## 3. Results

A total of 460 out of 1,102 facilities replied within four weeks, the response rate being 41.7%. According to the clients' main disabilities, each facility was classified into the following categories: ID, PDD, PIMD, physical disability group, and others (Table 1). The last group was small in number and diverse in character and also included responders who provided incomplete information; thus, data from this group were omitted from analysis hereafter. Also in Table 1, the number of clients (mean persons per facility) as an index of facility scale, their mean age, and proportion of male gender are shown.

The service providers who actually replied to the questionnaire were well trained and experienced. Many were working for over 20 years. Their average age (SD) was 44.7 (9.5) years old for the ID group, 53.0 (7.6) for the PDD group, 48.3 (8.4) for the PIMD group, and 55.3 (4.9) for the physical disability group. Their professions were diverse. Among the responders, there were 176 (38.3%) physicians, nurses, or psychologists. Many certified child care workers also responded.

373 out of the 415 facilities (about 90%) experienced cases with difficulties when communicating with the clients' parents. The percentage was high regardless of the children's disabilities, being concretely highest in the PDD group (96.9%) and lowest in the physical disability group (86.7%). The service providers noticed that the difficulties seemed to be increasing. The proportion of such parents, out of the total, was reported to be “almost none” (5% or less) or “few” (less than one fourth of the clients) in most of the facilities (Table 2). It should be noted that in the ID and PDD groups, about 15% of the facilities responded that “about half” or more of the parents were difficult to communicate with. In all groups, these difficulties were experienced particularly with the mothers, followed by the fathers. The characteristics of the ID and PDD groups were similar to each other, while the PIMD and physical disability groups resembled each other. In other words, in the ID and PDD groups, slightly more difficulties were experienced with the fathers compared to in the PIMD and physical disability groups, where they experienced more difficulties with other family members such as the grandparents and siblings compared to the ID and PDD groups.

The primary reason for these difficulties was thought to be the parent's personal condition, such as poor mental health (Table 2). This was especially true for the ID and PDD groups [*χ* ^2^(df = 9) = 41.9, *P* < 0.001]. In the PIMD and physical disability groups, the parent's poor socio-economic status and heavy care-giving burden, reflecting the child's level of disability, were considered to be major factors in approximately one-third of the cases.

Signs of depression or a depressive state were the most common psychiatric disturbances seen in the parents. Among the four groups, the PDD group most commonly experienced cases which showed signs of depression or a depressive state in parents. The proportion of such parents was approximately 70% of the total [*χ* ^2^(df = 12) = 65.1, *P* < 0.001]. Well-treated cases were only 5% out of the total; thus, most of these conditions were considered to be not medically well treated.

For further analysis, we selected 159 cases where symptoms of depression or a depressive state were reported in a descriptive manner. According to these reports, mainly the mothers suffered from this condition. We asked about the time of the onset, that is, whether it was prior to the birth of the child, when the parent first noticed the child's disability, or after the child had been fully medically diagnosed. Apart from the answer “unknown” which accounted for approximately half of the cases, “prior to the birth of the child” was relatively common in the PDD group (22.1%), whereas “after the child had been fully medically diagnosed” was relatively common in the PIMD (23.5%) and physical disability groups (28.6%).

When a parent suffered from signs of psychiatric disturbances, more than half of the children experienced maltreatment. The details of inappropriate treatment were analyzed (Table 3). Physical neglect was most common in all groups, accounting for about 50% of the cases. In comparison with the other groups, in the PDD group, the child was likely to be emotionally disturbed and confined indoors (32.6%), in the PIMD group, the child was mostly confined indoors (33.3%), and in the physical disability group, medical care neglect made the utilization of medical and welfare support impossible (50%) [*χ* ^2^(df = 12) = 27.6, *P* = 0.006].

Various measures were taken to deal with these difficult cases (Table 4). The leading measures were gathering and disclosure of necessary information, discussion among service providers within the facility about ways to deal with the parent, protection of the child by providing a safe environment, making approaches to other family members for cooperation, intensive collaboration with other organizations such as institutions and consultation centers for better community support, and medical support for the parent provided by specialists such as physicians and psychologists.

Many factors were reported to affect the mental health of parents. The presence or absence of support from other family members (i.e., a spouse) and the existence (or not) of a medical condition in the parent were major factors (Table 5). Practical use (or not) of social support networks, whether the child's level of disability was severe or not, and parent participation in face-to-face family support groups or not were also thought to be influential factors. In all groups, regardless of the child's disability, more direct welfare support for the child was considered to be helpful and thus requested to be improved, in order to meet the mental health needs of the parent. Besides this, in the physical disability group, family support groups, in the PIMD group, support (including financial support) for other family members, and in the ID and PDD groups, support for other family members (mainly siblings and the other parent) and medical treatment of the parent's psychiatric disturbance were also emphasized.

To fulfill the demanded future role of their facility, respondents expressed the importance of more collaboration with other institutions and consultation centers and the necessity for more special training courses targeting support professionals and recognized the urgent demand for more specialists especially psychologists who can support the mental health needs of both the children and their parents.

## 4. Discussion

In the present study, we surveyed institutions and consultation centers for children with developmental disabilities in Japan, which play a central role in the social support networks. This facility-based nationwide survey revealed the situation facing the parents of children with ID and other developmental disabilities from the professional caregivers' point of view. Almost all of the facilities experienced difficulties in keeping good relations with the clients' parents, regardless of the children's disabilities, and the majority of service providers noted that the proportion of such parents, out of the total, was roughly between 5 and 25%, and still increasing. The primary reason for these difficulties was thought to be the parent's condition, such as their mental health and background. Also, signs of depression or a depressive state were assumed to be the most common psychiatric disturbance seen. These results suggested that thorough consideration of the parents' mental health needs, especially prevention of depressive conditions and support for parents suffering from them, is urgent-demanded task for facilities.

ID, attention-deficit/hyperactivity disorder (AD/HD), and PDD are common developmental disorders, and the latter two are noted to have substantial genetic components [17]. It has been reported that a lifetime prevalence of major mood disorders is higher in parents of children with autism and that the onset for the majority was prior to the birth of their child with autism [18]. Family history studies of autism consistently uncovered a large subgroup with a high incidence of major mood disorders among family members, suggesting the two entities are related clinically and genetically [19, 20].

The findings of the present survey must be interpreted allowing for the limitations of the use of unconfirmed data reported by professional caregivers' who come into contact with children as their clients, and their parents on a day-to-day basis. Evidently, the service providers' perspective on parental mental health was consistent with previous reports from Western countries on major mood disorders and PDD. In other words, facilities where the clients were diagnosed as having PDD experienced significantly more cases of parents with signs of a depressive state compared to other facilities, and the onset of depression was considered to be prior to the birth of the child in many of the cases.

Although these professional caregivers were well trained and well experienced, only, one-third were physicians, nurses or psychologists. On account of the limitations of these data, we emphasize confirming the diagnoses of psychiatric disturbances through direct contact with the parents by specialists such as psychiatrists is necessary in future studies, for accurate understanding of the overall problem. In Japan, there is no official system to check and document the parents' condition including their mental health status. Parents are not accustomed to being asked about their own mental health, so there is a tendency to avoid discussion on matters concerning themselves. This was the major reason why we asked the professional caregivers about the parents' condition, not the parents directly, in this study. But still we judged it to be worthwhile at this moment to summarize the professional caregivers' impression on this issue, since the case in Japan has never been described in detail despite of its importance.

Because the majority of burden for daily care of handicapped children is typically carried by mothers, particular concern is raised for their adaptation. In the past studies, the psychosocial outcomes in mothers were said to be better predicted by psychosocial factors such as more active social life and family resources [21]. Recent research focusing on parenting stress illustrated the value of the participation of fathers also. Greater marital quality predicted lower parenting stress for both mothers and fathers, while greater social support predicted increased parenting efficacy for fathers [22]. Although the relationship between child characteristics and parental well-being has not reached an invariable agreement, the severity of a child's disability, intellectual functioning, and so forth are generally considered to be risk factors.

Since the burden for daily care is mainly carried by the mother, similarly in Japan, service providers tend to be in contact with the mother more frequently than other family members [23]. This may be partially the reason why service providers experienced difficulties in communicating with mostly the mothers in this study. The presence of support from other family members, such as a spouse, is considered to be a major influential factor for the mental health of parents in Japan also. It is presumed that greater marital quality is being reflected here.

Practical use of social support networks such as participation in face-to-face family support groups, more financial support for the household, more direct welfare support for the child, and medical treatment of the parent's psychiatric disturbance were reported to be helpful and thus requested to be improved, in order to meet the mental health needs of the parent. We would like to emphasize the fact that although the importance of medical treatment for the parents' psychiatric disturbances was pointed out, it was also realized that these conditions were presently not being medically well treated. This shows that current psychiatric services in Japan are not adequate to meet the complex mental health needs of the parents appropriately. To resolve this challenging issue, more intensive collaboration between other child-care facilities and organizations, aiming at the mental health needs of the parents, is demanded. The paucity of trained psychiatrists and other allied professionals also needs to be addressed, and in order to fulfill this, sufficient discussion and research on the establishment of an effective training system for specialists is warranted.

Providing direct advice for the parents on the utilization of social support networks is a reliable measure that can be immediately taken. Training more professionals who can properly deal with the parents' mental health needs is an urgent matter that must be tackled. More direct welfare support for the children is naturally helpful. Overall, the present study is the first nationwide survey that examined the professional caregivers' perspective on the mental health of parents of children with developmental disabilities, and it underscores the importance of understanding and supporting the parents' mental health needs, which leads to more appropriate support for children with developmental disabilities.

## Figures and Tables

**Table 1:** Descriptive information on each facility group.

Facilities (*n* = 460)		Client variables (*n* = 21, 017)		
Group	Number (%)	Mean persons per facility (SD)	Mean age (SD)	Male gender (%)
ID	201 (43.7)	39.6 (23.3)	15.3 (10.0)	70.7
PDD	113 (24.6)	41.0 (36.8)	6.2 (5.4)	74.0
PIMD	64 (13.9)	72.2 (41.7)	32.5 (11.4)	54.6
Physical disabilities	37 (8.0)	36.9 (24.9)	10.7 (10.1)	58.6
Others	45 (9.8)	—	—	—

Intellectual disability (ID), pervasive developmental disorder (PDD), profound intellectual and multiple disabilities (PIMD).

**Table 2:** Descriptive information on communication difficulties with parents.

	Group			
	ID	PDD	PIMD	Physical disabilities
Frequency of difficulties (%)				
Increasing	59.1	65.2	45.9	54.1
No change	34.2	22.3	34.4	24.3
Decreasing	2.1	3.6	3.3	0.0
Unknown	4.7	8.9	16.4	21.6
Ratio of difficult parents out of the total (%)				
Almost none (<5%)	31.5	28.8	49.2	42.9
Few (5–25%)	53.3	50.5	46.0	45.7
About half	11.7	10.8	4.8	5.7
Many (75–95%)	2.0	6.3	0.0	0.0
Almost all (>95%)	1.0	0.9	0.0	0.0
Unknown	0.5	2.7	0.0	5.7
Who was difficult (%)				
Mother	85.3	86.0	80.4	81.8
Father	8.7	9.3	7.1	6.1
Both mother and father	4.3	4.7	7.1	6.1
Others	1.6	0.0	5.4	6.1
Factors observed in the background of difficulties (%)				
Heavy care-giving burden	7.2	2.9	10.0	9.1
Poor socioeconomical status	10.0	6.7	36.7	18.2
Poor mental health/background	82.2	89.5	53.3	69.7
Others	0.6	1.0	0.0	3.0

Intellectual disability (ID), pervasive developmental disorder (PDD), profound intellectual and multiple disabilities (PIMD).

**Table 3:** Opinion on whether or not the child was inappropriately treated and the details of inappropriate treatment.

	Group			
	ID	PDD	PIMD	Physical disabilities
Whether or not inappropriately treated (%)				
Yes	56.3	52.4	35.3	66.7
On balance	22.9	30.5	35.3	16.7
No	12.5	9.8	17.6	0.0
Unknown	8.3	7.3	11.8	16.7
Details of inappropriate treatment (%)				
Physical neglect	55.6	46.5	66.7	25.0
Child confined indoors	11.1	16.3	33.3	25.0
Medical care neglect	0.0	9.3	0.0	50.0
Emotional neglect resulting in behavioral problems	0.0	16.3	0.0	0.0
Others	33.3	11.6	0.0	0.0

Intellectual disabilities (ID), pervasive developmental disorder (PDD), profound intellectual and multiple disabilities (PIMD).

**Table 4:** Most common measures taken when dealing with parents with signs of psychiatric disturbances.

	group			
	ID	PDD	PIMD	Physical disabilities
Taken measures (%)				
Close team work within the facility	36.1	21.6	26.1	60.0
Team work with other facilities	30.6	28.4	17.4	10.0
Support for the parent (by physicians)	6.9	11.4	8.7	0.0
Support for the parent (by psychologists)	2.8	13.6	13.0	20.0
Parent participation in family support groups	0.0	2.3	0.0	0.0
Protection of the child	9.7	11.4	8.7	0.0
Approach other family members	4.2	4.5	0.0	0.0
Nothing could be done	9.7	6.8	26.1	10.0

Intellectual disabilities (ID), pervasive developmental disorder (PDD), profound intellectual and multiple disabilities (PIMD).

**Table 5:** Influential factors and needs for the promotion of mental health in parents.

	Group			
	ID	PDD	PIMD	Physical disabilities
The factor most affecting mental health (%)				
Support from other family members	50.0	51.7	54.7	56.7
Existence of ill conditions in the parent	31.8	24.1	37.7	23.3
Practical use of social support networks	6.8	6.9	0.0	10.0
Child's level of disability	5.5	6.9	0.0	3.3
Parent participation in family support groups	2.5	6.9	1.9	3.3
Others	3.4	3.4	5.7	3.3
Helpful support which needs to be strengthened most (%)				
Direct welfare support for the child	39.7	38.9	32.8	48.5
Participation in family support groups	11.1	18.6	13.1	30.3
Support for other family members	25.9	27.4	31.1	6.1
Financial support for the household	4.8	0.0	13.1	0.0
Medical treatment of the parent's psychiatric disturbance	13.2	9.7	4.9	6.1
Others	5.3	5.3	4.9	9.1
Demands which facilites are most likely to face (%)				
More close team work with other facilities	43.8	42.9	37.3	43.8
More training courses for support professionals	33.2	36.2	31.3	31.3
Resolving the paucity of trained professionals	17.1	17.9	21.7	20.8
Others	5.8	3.1	9.6	4.2

Intellectual disability (ID), pervasive developmental disorder (PDD), profound intellectual and multiple disabilities (PIMD).
